# Chimerism-Based Experimental Models for Tolerance Induction in Vascularized Composite Allografts: Cleveland Clinic Research Experience

**DOI:** 10.1155/2013/831410

**Published:** 2013-03-14

**Authors:** Maria Siemionow, Aleksandra Klimczak

**Affiliations:** ^1^Department of Plastic Surgery, Cleveland Clinic, 9500 Euclid Avenue, A-60, Cleveland, OH 44195, USA; ^2^Institute of Immunology and Experimental Therapy, Polish Academy of Sciences, Rudolfa Weigla Street 12, 53-114 Wroclaw, Poland

## Abstract

The preclinical experimental models of vascularized composite allografts (VCAs) have been rapidly developed for the assessment of immunomodulatory protocols for clinical application. Recently, researchers have focused on immunomodulatory protocols which overcome the immunologic barrier between the allogeneic donor and recipient and may lead to tolerance induction. In order to test the feasibility of chimerism induction, experimental VCAs have been performed in different models including rodents, large animals, and nonhuman primates. These models differ in the complexity of transplanted tissue and in their responses to immunomodulatory protocols. In most applications, VCA contains multiple-tissue components; however, each individual component of CTA possesses unique immunologic characteristics that ultimately contribute to the chimerism induction and successful outcome of the VCA. Heterogenic character and complexity of tissue components in different VCA models determine the quality and robustness of donor-specific chimerism. As introduced in experimental studies, variable immunomodulatory options have been studied to achieve tolerance to VCA in rodents and large animal models allowing for widespread application in clinic. In this paper, based on our own experience, we have analyzed the current knowledge of tolerance-inducing strategies via chimerism induction in VCA experimental models in the context of immunomodulatory protocols and VCA complexity and their relevance and applicability to clinical practice.

## 1. Introduction

Experimental models of vascularized composite allografts (VCAs) successfully preceded clinical application of VCA, especially hand and face transplants, which have become a breakthrough in the fields of reconstruction for patients suffering from massive complex tissue injury. Although allotransplantation, as a reconstructive option, has become widely accepted as an experimental procedure in clinic, it still raises a lot of attention due to lifelong immunosuppression. To date, 59 hands in 41 patients and 24 partial or full face transplants, which are considered the most challenging VCAs, have been successfully performed in clinic (IRHCTT; http://www.handregistry.com/) [1, 2].

Experimental models of VCAs were created not only to assess the surgical feasibility and functional recovery after allotransplantation, but also to test tolerance-inducing strategies based on immunomodulatory protocols which will have potential application in clinic [3, 4]. Extensive research on tolerance induction performed during the last two decades has proven that development of donor-specific chimerism may accompany induction of tolerance in VCA; however, the role of chimerism in tolerance induction is still debatable [5–7]. 

Tissue resident cells, which are present within the transplanted tissue, may play an immunomodulatory role when the proper immunosuppressive regimen is applied. Immunocompetent cells present within the transplanted tissue are known as passenger leukocytes and, after vessel anastomosis between the transplanted VCA and recipient vessels, they may migrate into different compartments of the recipient and contribute to chimerism induction. The role of passenger leukocytes was confirmed by Starzl in his pioneered studies on the role of chimerism in solid organ acceptance [8].

The heterogenic character of tissue components in different VCA models determines the quality and robustness of donor-specific chimerism. A rodent MHC-mismatched model offers the advantage of identification of donor versus recipient cells, using monoclonal antibodies specific for MHC strains of rodents. 

Our own observations indicate that a universal tolerogenic protocol for VCA still does not exist, and the success of VCA acceptance depends on the immunologic character of transplanted tissues, their complexity, and the genetic barrier between donor and recipient. 

In this paper we analyze our experience and the current knowledge on tolerance via chimerism induction strategies in experimental VCA models. Immunomodulatory protocols used in experimental models include (i) monotherapy protocols using calcineurin inhibitors such as cyclosporine A (CsA) or tacrolimus, (ii) T-cell depletion protocol, and (iii) protocols augmented with donor bone marrow cells (BMCs). These protocols will be analyzed in the context of chimerism induction and VCA complexity. 

## 2. Monotherapy Protocol with Calcineurin Inhibitors for Chimerism Induction in VCA

Monotherapy protocol with CsA has been applied in many experimental VCA models including models with a single component of allograft (skin) and in more complex models such as limb and face allografts [9–23].

### 2.1. Vascularized Skin Allograft: The Model of a Single Tissue Component

Skin represents an important component of VCA and may be transplanted as a single component to cover large skin defects or as an integral part of composite tissue allograft including hand and face transplants.

Many immunocompetent cells, including Langerhans cells (LCs) and dermal dendritic cells (DDC), are present in the skin, both with an antigen-presenting function, as well as dermal T lymphocytes. The highly immunogenic character of skin represents a significant challenge for skin acceptance and an experimental skin model is the most frequently used model for tolerance induction studies [24]. 

In our experimental design of VCA, we have performed a study to determine if there is correlation between the vascularization and development of donor-specific chimerism in different sizes of vascularized skin allografts (VSAs) and nonvascularized skin allografts (NVSAs) in the rat model, under low-maintenance dose of CsA monotherapy (2 mg/kg/day) [9]. In this study, we have documented that vascularization and size of the skin allograft contribute to both skin allograft survival and donor chimerism induction. We observed the presence of donor chimerism in both vascularized and nonvascularized skin grafts; however, the dynamics and level of chimerism differed between transplanted groups. We have confirmed that larger graft size correlates positively with chimerism level, only in the VSA recipients, and initially, at one week posttransplant, chimerism was assessed at 12.2% in large skin allograft recipients (6 × 6 cm) versus 8.0% in the group receiving smaller (2 × 2 cm) skin allografts (*P* < 0.05) [9].

In contrast, in NVSA, recipient's larger skin diameter correlated inversely with blood chimerism level and at day 7 following-transplant; the mean value of total donor chimerism was assessed at 2.53% in the group receiving large (6 × 6 cm) skin grafts versus 3.92% in the group receiving small (2 × 2 cm) skin allografts (*P* < 0.05) [9]. 

In both types of skin transplants, VSA and NVSA, chimerism declined during the follow-up period, and two months after transplantation, it revealed levels of 1.1% to 1.6% in the VSA group and was found to be below 0.5% in the NVSA group. The level of chimerism correlated with allograft survival and skin vascularization. The differences in chimerism level in VSA when compared with NVSA are dependent upon the progress of allograft vascularization. After transplantation of VSA, blood supply returns to the allograft within 1-2 hours after donor-recipient vessel anastomosis and this minimizes ischemic as well as reperfusion-related damage. Moreover, graft-resident cells rapidly migrate into the recipient's blood circulation, which contributes to chimerism induction. In contrast, in NVSA transplants, graft revascularization takes at least a few days, and this extends relative ischemia time, with its known complications. During this early period, there is sprouting of new vessels from the recipient bed and neighboring recipient skin which are reaching the graft; thus, there is no direct connection between donor-origin cells from the graft and the recipient's immune system, via blood circulation, as is the case in VSA models. The smaller size of skin graft is more susceptible to revascularization and this may explain higher chimerism-level small-size allografts when compared to the larger-size NVSA. 

Our observation confirmed the dynamics of the skin allograft vascularization in non-VSA and VSA models, as well as graft size, to have a significant effect on the development of donor chimerism. 

Total abdominal wall (TAW) transplant in a rat model has been developed in our laboratory to monitor immunologic responses in the largest VSA transplant (8 ×12 cm^2^) [10]. This is the first model of large vascularized skin allograft transplant in a small animal, simulating a clinical abdominal wall transplantation with consistent anatomy, straightforward surgical technique, and reliable blood supply, which are essential for the success of experimental transplantation studies. The transplantation procedure was performed under a maintenance dose of CsA monotherapy started from 16 mg/kg/day and maintained at 2 mg/kg/day after 4 week posttransplant.

Chimerism levels were monitored and at day 7 posttransplant, the mean value of total chimerism was assessed at 6.7 ± 1.32% or the presence of donor-origin cells; however, over time, chimerism declined and at day 100 posttransplant revealed 1.3 ± 0.38%.

These studies on skin allograft transplants have proven that skin is an abundant source of donor-origin cells which are able to migrate and engraft to the recipient compartments, leading to chimerism induction and maintenance when supported by adequate immunosuppressive therapy.

### 2.2. Complexity of the VCA: The Multitissue Models

Complexity of the VCA introduces surgical and immunological challenges and requires adjustment of immunosuppressive protocols. In most clinical applications, such as hand and face transplants, VCA contains multitissue components including skin, subcutaneous tissue, muscle, bone with bone marrow, lymph nodes, nerve, tendon, and mucosa. The most commonly used experimental model of VCA is the orthotopic and heterotopic limb allograft transplant. 

#### 2.2.1. The Limb Allograft Model

The limb represents a specific model of the VCA since vascularized bone, with bone marrow cells, constitutes a structural component of the VCA in addition to muscles, skin, nerves, and tendons. We have shown that a limb allograft contains approximately 50 × 10^6^ of the bone marrow cells which may play a significant role in chimerism induction [11]. 

Experience with successful experimental limb transplantation across MHC-mismatched rat strains was reported by Kim et al. [12], where successful limb allograft survival was accomplished under a maintenance dose (10 mg/kg/day) of CsA monotherapy. Kim reported that continued CsA delivery is mandatory for limb allograft survival, since animals rejected transplanted limbs within 1 week following CsA cessation. However, Black et al. reported indefinite limb allograft survival under a moderate daily dose of CsA (8 mg/kg/day), given for 20 days posttransplant, followed by a maintenance dose of CsA given twice a week [13]. These studies proved that maintenance CsA therapy is essential for limb allograft survival.

Our experience with limb allograft model under continued CsA monotherapy resulted in long allograft survival [14]. In this study, semiallogenic rat hind-limb transplantations were performed under low-dose CsA protocol (4 mg/kg/day) combined with topical steroids, fluocinolone acetonide (6 mg/cm^2^/day), both started at the day of surgery and maintained during the entire follow-up period. Synergistic therapeutic effect of the low dose of CsA and topical application of steroids allowed for extended limb allograft survival, up to 51 days. 

The first studies reported by Kim, Black, and Inceoglu documented the technical feasibility and beneficial effect of CsA in limb VCA survival, but chimerism was not assessed in these studies.

Hewitt et al. reported hind-limb transplants between Lewis and Lewis × Brown-Norway (LBN) rats, in immunologically unmodified limb allograft recipients [15]. The authors documented that development of a high level of hematopoietic donor-specific chimerism of 60.2 ± 14.5% was associated with development of GvHD, whereas the presence of a stable, low level of mixed T-cell chimerism, below 18.3 ± 3.9%, was associated with tolerance induction in most of the limb allograft recipients (*P* < 0.002).

Several studies on limb allograft under the CsA protocol were also performed in a large animal model. Bourget et al. tested the effect of a 12-day course of CsA monotherapy (13 mg/kg/day) in MHC-matched, minor antigen-mismatched miniature swine model [16]. The authors reported long-term survival of the musculoskeletal component of limb allograft recipients under CsA monotherapy, and this was associated with the presence of transient chimerism which was detectable until day 19 posttransplant. Authors concluded that transient hematopoietic chimerism is sufficient for tolerance induction in the large-animal model of VCA [16]. 

#### 2.2.2. Face Allograft Model

Face allograft is an example of the most complex VCA models and may be transplanted with or without a vascularized bone component.

(1) *Face Allograft without Bone Component*. The first full face/scalp allograft model was introduced in a rat, in the year 2000, by Siemionow et al., in the Microsurgery Laboratory of Cleveland Clinic. Since that time, Siemionow's team has developed different experimental models of rat face transplantation that differ in their content of transplanted tissue and immunosuppressive protocols. In 2003, first reports that documented successful face/scalp allograft survival between LBN donors and Lewis recipients under CsA monotherapy (16 mg/kg/day), tapered within four weeks to low maintenance dose of 2 mg/kg/day, were introduced [17]. Following full face transplantation, we developed a hemiface transplant model to test the feasibility of tolerance induction and immunological response to different protocols [18]. The immunosuppressive protocol of CsA maintenance monotherapy (2 mg/kg/day) was tested in semiallogenic (LBN to Lewis) and fully MHC-mismatched (ACI to Lewis) models, corresponding to a more stringent and clinically relevant scenario [19]. Long-term survival in both models was associated with the presence of donor-specific chimerism in both T-cell and B-cell lineages, assessed both in the peripheral blood and bone marrow compartments, and was associated with engraftment of donor-origin cells to lymphoid organs of recipients. In semiallogenic hemiface model, T-cell and B-cell chimerisms were assessed at 10.14% for the CD4 and at 6.38% for CD8 T-cell population and at 10.02% for B-cell lineage represented by CD45RA antigen. In complete MHC-mismatched (ACI to Lewis) face transplant model, a high level of donor chimerism was detected (17.54% for CD4 and 9.28% for CD8) in T-cell population; however, low chimerism (below 1%) was assessed for B lymphocytes. Moreover, we have confirmed the engraftment of cells of allograft origin into spleen and lymph nodes, but not to the thymus, of the face transplant recipients [19]. 

Development of chimerism in a face allograft model may be explained by the rich representation of dermal T lymphocytes within skin component, as well as lymph nodes which are an abundant source of donor T and B cells.

(2) *Face Allograft Model with Bone Component*. The clinical need to cover extensive craniomaxillofacial defects, including bony and soft tissue components, encouraged us to develop rat model of composite hemiface/calvaria, maxilla, and hemiface/mandible/tongue transplantation models [20–23]. These surgically challenging models were maintained under low nontoxic dose of CsA (2 mg/kg/day) monotherapy and immunologically assessed for the presence of chimerism at different time points starting from day 7 posttransplant with the end-point at the sacrifice day. 

In hemiface/calvaria model, viable bone marrow cells were detected within vascularized bone component, and peripheral blood chimerism was supported predominantly by B-lymphocyte population.

 In a heterotopic rat maxilla model, which contains only bone and mucosal tissue (without skin), donor chimerism was detectable in long-term survivals (over 100 days posttransplant) and was represented by CD4 (12.5%) and CD8 (5.3%) T lymphocytes and by 4.7% of B lymphocytes [21]. 

The purpose of developing an orthotopic composite hemiface/mandible/tongue model was to extend application of our standard face transplantation model in the rat by incorporation of the vascularized mandible, masseter, and tongue; to test its feasibility across the MHC barrier; and to assess the immunomodulatory effect of different tissue components of hemiface/mandible/tongue allograft and their contribution to the development and maintenance of multilineage chimerism [23]. 

Under CsA monotherapy, chimerism was initially characterized by a high level of donor-origin T cells assessed at 12.3% for CD4 and at 11.3% for CD8 T-lymphocyte subpopulations, whereas B-cell chimerism was lower (2.8%), assessed for CD45RA B-cell-specific antigen. Chimerism kinetics switched over time and T-cell chimerism declined, whereas B-cell chimerism at day 300 posttransplant was maintained and was assessed at 4.4%. Donor-origin cells were also detected in the bone marrow compartment of hemiface/mandible/tongue recipients, at 2.33%, and 1.21% of total chimerism was represented by immature RT1^n^/CD90+ cell phenotype [23].

In maxilla and hemiface/mandible/tongue models, the oral mucosa contains submaxillary and submandibular lymph nodes and salivary glands. Salivary glands contain a diverse population of lymphocytes represented by T cells, B cells, and natural killer cells. These cells are distinct from cells present in peripheral lymphoid organs and are known to be responsible for regulation and mediation of humoral and cellular immune responses in the mucosal immune network [25].

These findings indicate that bone marrow, lymphoid, and glandular components of the hemiface/calvaria, maxilla, and composite hemiface/mandible/tongue allograft have a positive immunomodulatory effect supporting development of donor chimerism and long-term allograft survival [26]. Maintaining a balance between chimerism induction and maintenance is crucial for long-term survival of facial VCA in a rat model.

Recently, therapy with tacrolimus and mycophenolate mofetil (MMF) was introduced in large-animal model of heterotopical facial VCA in nonhuman primates [26]. The heterotopically transplanted facial segment contained vascularized bone marrow (VBM) contained within donor mandible. Facial allograft recipients were maintained on tacrolimus (blood level 15–25 ng/mL) and MMF (50 mg/kg/day). Recipients of the facial allograft with VBM component demonstrated prolonged allograft survival when under maintenance immunosuppression; however, discontinuation of immunosuppression resulted in facial allograft rejection. Facial VCAs without bone component were rejected within 7–15 days despite continuous immunosuppression. Macrochimerism was detectable in both groups in blood and peripheral lymphoid tissues, spleen, and lymph nodes. These observations support the immunomodulatory role of hematopoietic cells present within VCA that facilitate stable graft acceptance with a modest requirement for immunosuppression [26].

## 3. The Role of Combined T-Cell Depletion and Immunosuppression in Chimerism Induction

Elimination of memory T lymphocytes or inhibition of T-cell activation represents a critical mechanism in the induction of transplantation tolerance [27]. Currently, immunodepletive protocols are widely used as part of an immunosuppressive regimen, both in clinic and in experimental models. Nonselective depletion of T cells is accomplished by either polyclonal anti-lymphocyte serum (ALS), anti-thymocyte globulin (ATG), or monoclonal antibodies such as anti-CD3 (muromonab-CD3) and anti-CD52 (Campath-1H) antibody. In contrast, selective depletion of specific populations of T lymphocytes eliminates only alloreactive T cells [28]. When recipients are submitted to depletive protocols, they are protected against graft-versus-host disease (GvHD), since immunodepletive agents eliminate graft-derived alloreactive T cells. 

In our experiments with chimerism induction under immunodepletive protocols, we have used ALS and anti-*αβ*-TCR monoclonal antibodies (anti-*αβ*-TCR mAb) to achieve tolerance.

**Figure 1:**
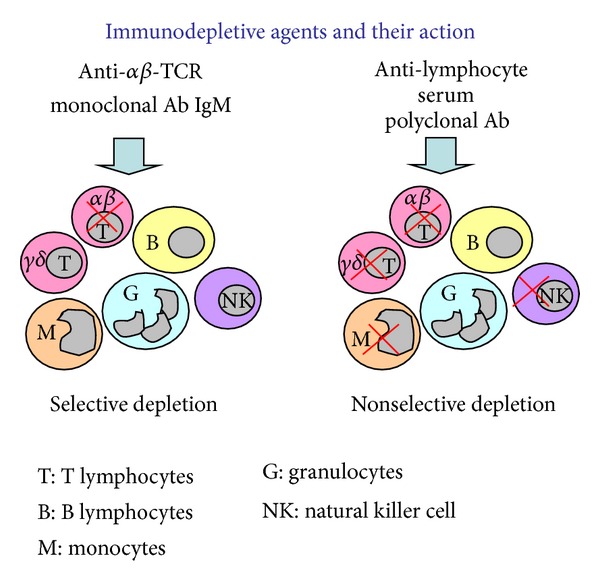
Selective immunodepletion under anti-*αβ*-TCR monoclonal antibody and nonselective depletion of leukocytes under anti-lymphocyte serum.

The polyclonal nature of ALS results in diverse immunosuppressive effects. ALS successfully eliminates all subpopulations of T lymphocytes (Figure 1) in peripheral blood and tissues via cytotoxicity and/or opsonization [29]. Moreover, ALS mediates leukocyte/endothelial level interactions by modulation of adhesion molecules or chemokine receptor expression. The immunomodulatory activity of ALS is also accomplished by interference with dendritic cell function; ALS acts as costimulatory blocker inhibiting maturation of dendritic cells and reduces the stimulatory capacity of dendritic cells for T-cell proliferation. In addition, ALS substantially depletes blood monocytes and NK cells, and this diminishes their innate immunity, contributing to prevention of allograft rejection, in addition to T-cell depletion. This action may, however, lead to development of opportunistic infections. 

In contrast, by selective depletion with anti-*αβ*-TCR mAb, only alloreactive T cells are targeted by specific inhibition of *αβ*-TCR, but other cells such as *γδ* T cells, natural killer (NK) cells, monocytes, and other leukocytes are preserved [30] (Figure 1). The *αβ*-TCR is expressed on the vast majority of immature and mature rat T lymphocytes and is responsible for the first signal of T-cell activation. By inhibiting the first signal of T-cell activation with anti-*αβ*-TCR mAb, alloreactive T cells, which are the main players of acute rejection, are selectively eliminated leading to peripheral anergy. The anti-*αβ*-TCR mAb acts as a depleting agent on target cells; however, functional inhibition has also been reported [31]. Immunocytochemistry has confirmed reduced TCR intensity staining after anti-*αβ*-TCR mAb therapy, both in our studies and the reports of other investigators [32, 33]. Moreover, this antibody is not mitogenic and initiates low first dose of cytokine release as compared to some other anti-T-cell monoclonal antibodies [34]. In addition, anti-*αβ*-TCR therapy downregulates endothelial activation and expression of many proinflammatory cytokines (e.g., IL-2 and IFN-*γ*) which are associated with allorecognition and development of rejection, as confirmed in the rat model of cardiac allografts [31, 35]. 

Immunodepletive agents are not that effective in tolerance induction when administered alone, but when induction therapy with immunodepletive agents is supported with short-term immunosuppression, irradiation, or costimulatory blockade, this type of protocol represents a powerful tool for chimerism development and tolerance induction.

### 3.1. Immunodepletive Protocols in the Limb Allograft Model

We have investigated tolerance induction in a limb allograft model, using a 21-day combined protocol of ALS and CsA therapy. Transplantations were performed in semiallogenic rat model between LBN (RT1^*l*+*n*^) donors and Lewis (RT1^*l*^) recipients. The combined immunodepletive protocol of ALS and CsA significantly prolonged limb allograft survival (over 420 days) compared to monotherapy with ALS or CsA alone (6 and 23 days, resp.), and tolerance was confirmed *ex vivo* by MLR assay showing hyporesponsiveness to the donor antigens and *in vivo* by acceptance of donor skin grafts. In addition, at 100 days posttransplant, immunocompetence of the recipients was confirmed by rejection of the third-party skin allograft. Tolerant animals demonstrated a donor-specific hematopoietic chimerism in the peripheral blood ranging from 35% to 42%, whereas in nontolerant animals chimerism was not detected [36].

After achieving success in tolerance induction in a semiallogenic limb transplant model, we applied the immunodepletive protocol of ALS and CsA to a more immunogenetically challenging model in fully MHC-mismatched animals (BN(RT1^*n*^) donors and Lewis (RT1^*l*^) recipients. Under the ALS/CsA protocol, limb allograft survival was extended by up to 56 days; however, tolerance was not achieved [37]. Only transient, donor-derived chimerism (17 ± 1.1% at day 35) was detected and dropped down to 0 at the time of rejection. This study confirmed that transplantation across a strong MHC barrier mandates adjustments in immunosuppressive protocols.

**Figure 2:**
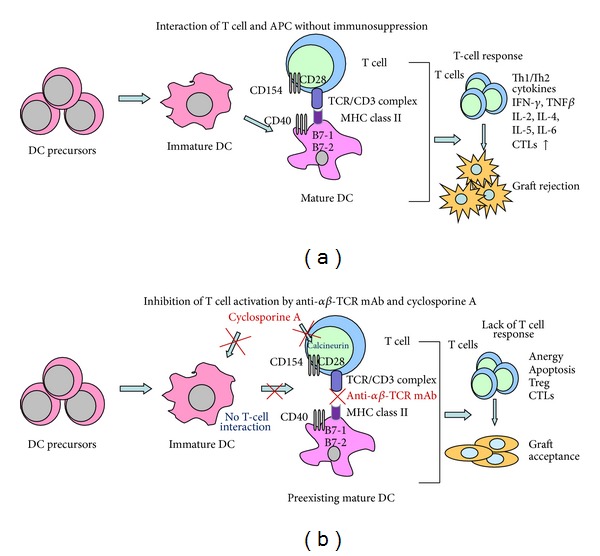
(a) Interaction of memory T cells and antigen pretention cells (APCs) without immunosuppression induced T-cell response and allograft rejection. (b) Selective targeting of *αβ*-TCR of TCR/CD3 complex inhibits the first signal of T-cell activation. Inhibition of immune response is enhanced by CsA, which inhibits IL-2 production by T cells and reduces expression of costimulatory molecules of dendritic cells. Lack of immune response by T cells facilitates allograft acceptance.

The success of tolerance induction in a limb allograft model under combined ALS and CsA therapy encouraged us to develop a new protocol of selective inhibition of potentially alloreactive *αβ*-TCR T cells, in combination with a short course of CsA therapy (Figure 2). Initial studies tested the dose and duration of anti-*αβ*-TCR mAb CsA therapy and resulted in establishment of dose of anti-*αβ*-TCR monoclonal antibody, at 50 *μ*g/day, in combination with tapered dose of CsA, from 16 mg/kg/day to 2 mg/kg/day, over 35-days posttransplant under this protocol [38]. Limb allograft survival (over 720 days) was associated with the presence of donor-specific chimerism in CD4 (6.7%) and CD8 (1.2%) T-cell subpopulation. Tolerance to alloantigens was confirmed *in vivo* by acceptance of the donor skin graft, and the immune competence of recipients was confirmed by rejection of third-party grafts. In contrast, a 35-day protocol of CsA monotherapy resulted in limb allograft rejection within two weeks after cessation of immunosuppression.

To further test the efficacy of short-term anti-*αβ*-TCR/CsA protocol, we investigated the effect of 21-, 7-, and 5-day protocols for chimerism development, allograft survival, and tolerance induction [39]. Indefinite limb allograft survival and functional recovery were associated with the presence of a stable level of donor-specific chimerism ranging from 10 to 12% in CD4 and 6 to 9% in CD8 T-cell subpopulation. Tolerance to donor antigens was confirmed *in vivo *by skin grafting and immunocompetence was confirmed by MLR assay. In this study, a combined anti-*αβ*-TCR/CsA protocol resulted in over 95% depletion of *αβ*-TCR-positive cells at, as early as, posttransplant day 7, and T-cell repopulation was present at 35 days after treatment cessation. The timing of deletional effect under 5-day protocol correlates with the maturation process of newly developed T cells (both from the donor and the recipient) in thymus, which takes approximately 28 days, and thus the short period of immunodepletion is sufficient to create a chronological window of unresponsiveness to the new repertoire of T lymphocytes [39]. We have confirmed that 5-, 7-, and 21-day immunodepletive protocols with anti-*αβ*-TCR/CsA resulted in long-term limb allograft survival, and we have chosen 7-day therapy as a standard immunodepletive protocol for tolerance induction in VCA. The rationale to choose 7-day protocol of *αβ*-TCR/CsA is the opportunity to use this protocol at the day of transplantation without recipient preconditioning and this has the advantages of direct clinical application since in clinical VCA a preconditioning protocol rather will never be accepted. 

Central (intrathymic) clonal deletion provides a robust form of tolerance in all chimerism-related approaches, even to the most immunogenic tissue, such as skin. Clonal deletion is usually considered superior to regulatory or anergic mechanisms since clonal deletion physically eliminates T cells with certain specificity [7]. To assess the role of thymus in tolerance induction in VCA, a series of experiments were designed using 7-day combined immunosuppressive protocol of CsA with T-cell depletion using anti-*αβ*-TCR/CsA in rat limb allograft model [33]. Allotransplants were performed between semiallogenic LBN donors and euthymic and thymectomized Lewis rat recipients without maintenance therapy. Treatment with *αβ*-TCR/CsA resulted in indefinite limb allograft survival (median survival time = 370 days) in euthymic recipients; however, a combined protocol of anti-*αβ*-TCR/CsA applied to thymectomized Lewis recipients should cover 51 days, the median survival time (MST) of limb allografts. In contrast, in control monotherapy groups with *αβ*-TCR or CsA in euthymic Lewis recipients, the MST of limb allografts was 13 and 22 days, respectively.

Stable T-cell chimerism of donor origin was achieved at 17.3% for CD4 and at 13.9% for CD8, in euthymic rats, whereas only transient chimerism, 7%–9% for CD4 and 2%–4% for CD8 T cells, was detected in the thymectomized rats. Immunoperoxidase staining confirmed engraftment of donor-origin cells into lymphoid organs (spleen, lymph nodes, and thymus) of the recipients in the euthymic rats under anti-*αβ*-TCR/CsA protocol. The morphology of many of the engrafted cells resembled that of dendritic cells. In contrast, in thymectomized limb allograft recipients, donor-origin cells were detected in the spleen and lymph nodes at the time of anti-*αβ*-TCR/CsA immunosuppressive protocol cessation but were absent in the lymph nodes, and only scattered cells were found in the spleen, at the time of allograft rejection. 

This study confirmed that mixed chimerism ensures intrathymic T-cell deletion of donor-reactive cells, as long as chimerism persists. This is mediated mainly by bone-marrow-derived dendritic cells of both donor and recipient origins. In this limb allograft model, the constant delivery of bone marrow cells of donor origin was permitted from the transplanted limb containing both the femoral and tibial bones containing hematopoietic cells. Mixed chimerism provides cells with an antigen-presenting function of both donor and recipient acting in the periphery and preserving recipient's immunocompetence to the third party antigens. In our experimental limb allograft model, MLR assay and skin grafting confirmed donor-specific tolerance in euthymic limb allograft recipients. Based on these observations, the authors suggest that the nonmyeloablative 7-day protocol of selective targeting of *αβ*-TCR-positive cells, in combination with CsA therapy, may facilitate engraftment of donor cells into the thymus, leading to negative selection of newly developing alloreactive host T cells. Both a central and peripheral mechanism may be involved in chimerism maintenance and tolerance to limb allograft. 

A successful protocol of combined anti-*αβ*-TCR/CsA with selective depletion of potentially alloreactive T cells was also applied in a fully MHC-mismatched rat limb allograft model, making this short-term, nonmyeloablative VCA conditioning, clinically applicable. Tolerance to the limb allograft was associated with stable, multilineage, donor-specific chimerism in the T-cell population: CD4 (7.6%) and CD8 (1.3%), and chimerism maintenance was supported by the B-cell lineage (16.5% of RT1^*n*^/CD45RA) [40]. 

In all limb allograft models, a vascularized bone component containing bone marrow cells of donor origin contributed to long-term femur allograft survival. Following revascularization, bone marrow cells migrated from the VCA donor and engrafted and repopulated in different tissues of the limb recipients, including the recipient's bone marrow compartment and, in this way, contributed to chimerism maintenance. 

 Our experience with VCA models has confirmed that reliable and stable chimerism, particularly in T-cell population, is a critical component for successful tolerance induction in VCA models without bone component, whereas more robust chimerism, in B-cell lineage, contributes to long-term survival when VCA contains vascularized bone compartment with donor hematopoietic cells.

### 3.2. Immunodepletive Protocol for Single Components of VCA

To test the effect of nonmyeloablative selective depletion of alloreactive T cells and to evaluate the contribution of skin and bone with bone marrow cells (single components of limb and face VCA) to chimerism induction, we have developed models of vascularized skin allograft from the groin region (groin flaps) [41] and unilateral and bilateral vascularized femoral bone transplantation [42–44]. 

#### 3.2.1. Immunodepletive Protocol in the Vascularized Skin Allograft Model

Our other approach to tolerance induction via chimerism in VCA models was to test the efficacy of a short-term immunodepletive protocol using anti-*αβ*-TCR monoclonal antibody in combination with calcineurin inhibitors, either CsA or tacrolimus, in assessment of the vascularized skin allograft transplantation level [41]. The groin flap was used as an experimental model of VSA and was transplanted across full MHC barrier between ACI donor and Lewis recipients. In this model, immunosuppressive therapy was given for 7 days only, and the vascularized skin allograft was transplanted, without recipient conditioning. Under this protocol of anti-*αβ*-TCR/CsA, extension of skin allograft survival was observed up to 84 days posttransplant and was associated with the presence of donor chimerism of T-cell origin (4.7% in CD4 and 1.4% in CD8). Lifelong tolerance to the skin allograft was not confirmed; however, this observation indicates that the skin allograft, when transplanted alone, requires stronger immunosuppression than that when it constitutes a structural component of complex VCA, such as face or limb transplant [45].

The methods of manipulation of immune system which are applied for tolerance induction of vascularized skin components using donor hematopoietic cell transplantation and nonhematopoietic approaches, via T-cell depletion or costimulatory blockade, are reviewed by Horner et al. [24].

#### 3.2.2. Immunodepletive Protocol in Vascularized Bone Marrow Transplants (VBMTs) of a Single Component of VCA Containing Bone Marrow Cells

Experimental limb allograft and face transplant models carrying bone component containing bone marrow cells (BMCs) are examples of vascularized bone marrow transplants (VBMTs). These models function as vascularized carrier of donor BMC, providing a continuous source of donor hematopoietic cell delivery, and are contributing to chimerism development and maintenance [46, 47]. 

The contribution of a vascularized bone marrow component in chimerism induction was investigated under our tolerogenic 7-day protocol of *αβ*-TCR mAb and CsA which was previously tested successfully in limb allograft transplants across an MHC barrier [44]. In this study, we documented that our protocol facilitated development of multilineage hematolymphoid chimerism via trafficking of the immature (CD90+) bone marrow cells (BMCs) between donor and recipient compartments. Early engraftment of donor BMCs into the recipient BM compartment was achieved at one week posttransplant and this was associated with active hematopoiesis within allografted bone and correlated with chimerism maintenance in the hematolymphoid organs in the thymus, spleen, and lymph nodes. Two-way trafficking between donor and recipient BM compartments was confirmed by presence of recipient MHC class I cells (RT1^*l*^ cells) within the allografted bone up to three weeks posttransplant. At ten weeks posttransplant, decline of BMC viability in allografted bone corresponded with bone fibrosis and lack of hematopoiesis, and further studies documented that this was associated with osteopontin overexpression [48]. In contrast, active hematopoiesis was present in the recipient bone with predominance of donor-specific, immature (CD90/RT1^*n*^) cells of B-cell lineage, which correlated with chimerism maintenance. The proliferative potential of donor-origin cells (RT1^*n*^) was confirmed by clonogenic activity confirmed *ex vivo* by colony forming units assay. These results confirm that hematolymphoid chimerism develops early after-VBMT and is supported by T-cell lineage and, despite allografted bone fibrosis, chimerism maintenance is supported by B-cell lineage and presence of active hematopoiesis of donor-origin cells in bone marrow environment of allograft host [44, 48].

To enhance chimerism induction and maintenance, a bilateral VBMT rat model was created [42, 43]. The kinetics of peripheral blood chimerism revealed that presence of donor-specific cells showed a peak at 3 weeks aftertransplantation. The chimerism was characterized by the prevalence of donor-origin B cells which ranged from 15.7% to 26.9% (mean 24.2%). In the bone marrow compartment, 28.2% of donor-derived cells were detected, and most of the donor-origin cells (24.1%) revealed an immature phenotype (CD90 + /RT1^*n*^+) which represents varying stages of bone marrow cell differentiation [43].

Two months after transplantation, peripheral blood chimerism declined to 1% for T lymphocytes and 1.5% for B lymphocytes, and these levels were maintained during the entire follow-up period of over 100 days posttransplant. In the host femoral bone marrow cavity, chimerism level was assessed at 10.4% and 3.7% of cells presented an immature phenotype of CD90 + /RT1^*n*^+ which was associated with maintenance of stable donor-specific chimerism [43].

The studies on VBMT tested under immunodepletive protocols have proven the beneficial effect of donor bone marrow cells for chimerism induction in VCA transplants containing bone component with viable hematopoietic cells of donor origin. The coexistence of donor and recipient hematopoietic cells within the recipient bone marrow compartment leads to lifelong mixed chimerism maintenance in all hematopoietic cell lineages and permits the lifetime presence of antigen-presenting cells of both the donor and recipient origins supporting tolerance to newly developed lymphocytes.

In clinical experience, vascularized bone constitutes a structural segment of hand, arm, and some of the complex face transplants. In humans, macrochimerism after VCA transplants has never been reported and only transient microchimerism has been detected in the early posttransplant period, both in hand [49] and face transplant recipients receiving donor BMC as part of the posttransplant therapeutic protocol [50].

## 4. Protocols Supported with Donor Origin-Hematopoietic Cells for Chimerism Induction

Hematopoietic chimerism was first introduced by Owen when Freemartin cattle (fraternal twins sharing a placental circulation) were shown to be chimeric and tolerant to each other [51]. Acquired tolerance to allogeneic skin via chimerism induction by hematopoietic cell transplantation into neonatal mice was first reported by Billingham et al. [52]. Since that time, different strategies for tolerance induction via donor BMT have been developed in experimental studies and in clinical practice [53–59].

 Vascularized bone with BMC was not yet clinically introduced as a supportive therapy of donor hematopoietic stem cells except in the cases where vascularized bone is an integral part of VCA (e.g., hand or face allograft). Based on the observation that VCA containing a bone with viable bone marrow compartment could function as a vascularized carrier of donor-origin bone marrow cells, providing a continuous source of donor hematopoietic cells, many experimental and clinical studies were developed for tolerance induction via chimerism. To test the beneficial effect of donor BMC for chimerism induction, we developed a new technique of intraosseous hematopoietic stem cell transplantation [53, 54]. 

In one study, we investigated the effect of intraosseous delivery of the selected population of donor-derived hematopoietic stem cell (HSC) CD90+ in rat hind-limb transplant model between Lewis-Brown-Norway and Lewis rats without immunosuppressive therapy [53]. Extended survival was achieved up to 15 days and was associated with 3.4% of donor-origin chimerism. In contrast, the control group without hematopoietic cell therapy rejected the limb allograft within 7 days [53]. 

The goal of donor BMT-based strategies for induction of transplant tolerance is to achieve the state when donor hematopoietic cells may reach the recipient thymus and promote negative selection of newly developed donor-reactive T cells [55]. We have tested, across the MHC barrier, the beneficial effect of intraosseous BMC delivery when compared with standard intravenous (i.v.) BMC transplantation. We discovered that hematopoietic recovery and efficacy of donor-cell engraftment into the BM and lymphoid organ compartments resulted in higher chimerism after intraosseous BMT (7.9% ± 1.3%) under *αβ*-TCR/CsA and 70 × 10^6^ BM cells, whereas lower chimerism (4.2% ± 1.4%) was observed after intravenous BMT [54]. The seeding efficacy of donor cells into lymphoid tissues, including thymus, was greater after intraosseous BMT when compared with standard i.v. transplantation (*P* = 0.007) [54]. 

 These observations indicate clinical applicability of a short-term immunodepletive protocol supported with donor bone marrow cells as a tolerance-inducing strategy in VCA. 

Recently, the role of donor bone marrow cells for chimerism induction was reported in a rat heterotopic osteomyocutaneous flap model transplanted to a mixed allogeneic chimera [56]. Mixed allogeneic chimeras were created 4 to 6 weeks before osteomyocutaneous flap transplantation. Rats were subjected to total body irradiation with 600 to 300 cGy and transplantation of 100 × 10^6^ T cells depleted with anti-*αβ*-TCR mAb bone marrow cells (day 0), followed by an 11-day course of tacrolimus and ALS (day 10) therapy. The long-term VCA survival was significantly better (57.1%) in chimeras receiving more than 300 cGy TBI and anti-*αβ*-TCR mAb as no long-term VCA acceptance was observed in animals treated with 300 cGy TBI without anti-*αβ*-TCR mAb preconditioning. Higher levels of chimerism, from 38.6% to 45.2%, were associated with VCA acceptance; however, the majority of flap acceptors lost peripheral blood chimerism within 6 months, but donor-origin cells were still present within the transplanted bone.

Clinical application of protocol utilizing hematopoietic cells for HLA-mismatched kidney transplantation underlines an immunologic benefit of donor bone marrow cells for transient chimerism induction and tolerance development to renal allograft [57]. However, this protocol requires conditioning therapies prior to donor BMT in order to induce chimerism and, clinically, is applicable only for living organ donors. A recent report introduced simultaneous kidney and bone marrow transplantation from 5 HLA haploidentical living-related donors under modified nonmyeloablative conditioning [58]. In all patients, transient multilineage chimerism was observed up to two weeks after transplantation, but rapid development of tolerance to the kidney allograft was achieved in one of these patients [58]. In the VCA protocol, pretransplant donor-specific chimerism creation will never be applicable in clinic; however, simultaneous or posttransplant supportive therapy with donor bone marrow cells is clinically relevant, as demonstrated by its introduction during the first face transplant performed in clinic [50]. 

To reduce maintenance immunosuppression, infusion of unmodified donor hematopoietic cells has been recently introduced for hand transplant recipients at the University of Pittsburgh [59]. Long-term clinical and immunological results of the Pittsburgh protocol are awaited.

## 5. Immunosuppressive versus Immunodepletive Protocols and Chimerism Induction

The differences in chimerism levels observed in rodents, large animals, and humans are based on biologic variation between the species and are attributable to their genetic and developmental differences, which can involve innate and adaptive immunologic function and metabolic responses to various treatment protocols [6]. 

CsA monotherapy protocol induces chimerism in all types of VCA; however, over time, chimerism declines and this is usually associated with allograft rejection. Moreover, discontinuation of calcineurin inhibitor monotherapy always leads to allograft rejection 2-3 weeks after immunosuppression withdrawal as confirmed in rodent and large-animal experimental models [12, 13, 26]. Immunosuppressive protocol with calcineurin inhibitors is associated with donor cell engraftment in the spleen and the lymph nodes but not in the thymus of the recipients, even when VBM was a part of VCA [11, 23, 60]. The lack of donor-origin cells in the thymus of CsA-treated VCA recipients may reflect CsA-mediated lymphokine downregulation, and disruption of thymic function, which is essential for cell-homing and engraftment [61]. CsA therapy induces changes within the thymic microenvironment leading to a reduction in the size of the thymic medulla, decreasing the number of interdigitating cells, and changing morphology of the epithelial cells [61]. All these changes limit donor cell engraftment and thymic chimerism development. Lack of thymic chimerism under CsA protocol prevents development of tolerogenic T cells among newly developing lymphocytes, and a low dose of CsA maintenance therapy is necessary to prevent alloreactivity and to maintain allograft survival. However, a low, nontoxic dose of CsA maintenance protocol, used in VCA, is permissive for “prope” tolerance induction as reported in solid organ transplantations [62]. The pharmacologic result is an altered immune response, inhibiting the activation process of T cells by IL-2 production and by downregulating surface costimulatory molecule expression on rodent and human dendritic cells [63, 64].

However, chronic immunosuppression with calcineurin inhibitors is associated with a risk of leucopenia, nephrotoxicity, or infectious complications. Based on tacrolimus monotherapy applied in a heterotopic face allograft study in nonhuman primates, rejection-free allograft survival ranging from 60 to 177 days was reported. A major limitation of this immunosuppressive approach was that 5 of 6 animals developed a posttransplant lymphoproliferative disease (PTLD) without clinical evidence of graft rejection [65].

When comparing the chimerism level of different VCA models performed under CsA protocol, VCA tissue complexity and its immunogenicity should be considered [66]. We found that the hemiface model presented the highest chimerism level when compared to the total abdominal wall or vascularized skin allograft models. Skin is a major component of all facial VCA and serves as an abundant source of donor immunocompetent cells which migrate into the recipient periphery. The face-and-neck region in rats is very rich in lymph nodes, and we suggest that the presence of lymph nodes within VCA contributes to a high chimerism level in the peripheral blood and lymphoid organs of recipients. Moreover, in the rat facial allograft model, donor-origin hematopoietic cells present in the vascularized bone of mandible or calvaria actively participate in chimerism induction. Finally, mucosal tissue, combined with salivary glands, in the face transplant model, is also a rich supply of donor-origin cells represented by T cells, B cells, and NK cells, which are distinct from the cells present in peripheral lymphoid organs and, after transplantation, may support chimerism induction and maintenance [21, 23]. Thus, facial VCA differs significantly from a total abdominal wall or vascularized skin allograft models since these allografts include only skin and subcutaneous fat tissue components but lack the mucosal component. We found that the level of chimerism in skin allograft models correlates proportionally with skin allograft dimensions [9]. 

Multilineage, mixed hematopoietic chimerism is associated with lifelong central, deletional T-cell tolerance, permitting acceptance of any allograft of donor origin without immunosuppression [67]. In limb allograft and VBMT models performed under an anti-*αβ*-TCR/CsA immunosuppressive protocol, we have observed engraftment of donor-origin cells into spleen, lymph nodes, and thymus [33, 44]. These observations suggest that a short-term immunodepletive protocol facilitates development of intrathymic microchimerism, which may be permissive for tolerance induction [33]. Moreover, the beneficial effect of selective depletion with anti-*αβ*-TCR mAb is accomplished due to the lack of cytokine-release syndrome after drug administration and faster T-cell recovery, which reduces the chance of development of complications inherent to immunodepletive agents [28]. 

The immunodepletive induction protocol with antithymocyte globulin (ATG), methylprednisolone, and maintenance therapy with FK506 and rapamycin was used for heterotopic transplantation of facial allografts in cynomolgus monkeys [68]. Under this protocol, long-term facial VCA survival ranging from 6 to 129 days posttransplant was achieved but tolerance was not induced, indicating that further development of immunosuppressive protocols is needed for nonhuman primate VCA models. In clinic, benefits of induction therapy with ATG or Campath-1 outweigh the adverse effects, especially when induction therapy is supported with calcineurin inhibitors or IL-2 signaling inhibitor [69]. However, these immunodepletive agents induce profound and durable lymphopenia that can be associated with adverse effects and immunodeficiency complications such as viral infections, CMV or EBV, or development of PTLD [70]. 

It is clear that none of the immunodepletive agents, neither nonselective nor selective T-cell depleters, are capable of acting as a single immunosuppressive agent. In our limb and face VCA models, induction therapy with an immunodepletive antibody, combined with CsA, significantly prolonged allograft survival and induced full or “prope” tolerance. Current experience with donor bone marrow transplantation, used as a supportive therapy in experimental VCA models, introduces viable strategies for tolerance induction which can be further refined and introduced to clinical cases of VCA such as hand or face transplants. The field of VCA transplantation is still open for introduction of innovative protocols such as our stem cell and chimeric cell therapies which have recently shown promising results in the face allograft model in rodents. These therapies may, in the near future, revolutionize the entire field of transplantation, including broad application of VCA.
